# Effect of Covid pandemic on immunization status of children in tertiary care Hospital of North India: reason for partial and non-immunization a cross-sectional study

**DOI:** 10.1186/s41043-023-00494-z

**Published:** 2024-01-15

**Authors:** Narender Kumar, Pinki Allyhan, Anju Aggarwal

**Affiliations:** Department of Pediatrics, University College of Medical Sciences and Guru Tegh Bahadur Hospital, New Delhi, 110095 India

**Keywords:** Immunization status, COVID-19 pandemic, Children

## Abstract

**Background:**

Low immunization coverage in India attributes to many factors including sociodemographic factors and people’s behavior. COVID-19 pandemic resulted in disruptions in achieving optimum availability and utilization of immunization services. This study was carried out to find out the immunization status of children in the post COVID era and various factors responsible for non-immunization during the pandemic.

**Methods:**

This cross-sectional study included parents of 225 admitted children aged 1–6 years were interviewed using a semi-structured open-ended questionnaire. Children were classified as completely immunized, partially immunized and unimmunized on the basis of vaccines missed given under first year of life. Reasons for non-immunization and delay/missed vaccination during COVID-19 pandemic were recorded.

**Results:**

Of the 225 children, 162 (72%; 95% CI 66–78%) were completely immunized, 55 (24.4%; 95% CI 19–30%) were partially immunized and 8 (3.6%; 95% CI 1–6%) were unimmunized. Parents with hospital deliveries, higher education level and lesser birth order were more likely to have children with better immunization status (*p* < 0.05). First dose of measles scheduled at 9 months and 3rd dose of pentavalent vaccine/OPV/Rotavirus vaccine scheduled at 14 weeks were most commonly missed vaccines among partially immunized. Lack of awareness (*n* = 36, 57.1%; 95% CI 45–70%) was the common reason for partial and non-immunization followed by illness of child (*n* = 21, 33.3%; 95% CI 21–45%) and COVID-19 pandemic (*n* = 11, 17.4%; 95% CI 8–27%). Pandemic was reason for delay in 50 (22.2%; 95% CI 17–28%) children. Restrictions of movement (64%; 95% CI 50–78%), fear of being exposed to COVID-19 (52%; 95% CI 38–66%) were the most common reasons for delay during the pandemic. Of the 50 children who had delay due to pandemic, 39 children (17.3%; 95% CI 12–22%) received their catch-up immunization after the pandemic. No child remained completely unimmunized due to COVID-19 pandemic.

**Conclusions:**

Although COVID-19 pandemic resulted in disruptions in routine immunization services, sociodemographic factors such as awareness for immunization, parental education and various beliefs for immunization were responsible for the children remaining unimmunized or partially immunized after the pandemic.

## Introduction

COVID-19 disease was officially declared as pandemic in March 2020 and lasted for about 2 years. Control measures included restriction on movements (lockdown) resulting in disruption of health care services including routine immunization. In May 2020, WHO reported nearly 90% disruptions to essential health services, immunization services being most frequently affected [1, 2]. Coverage of DPT-3 and first dose of measles containing vaccine (MCV-1) dropped from 86% in 2019 to 81% in 2021, leaving around 5 million more children unvaccinated in 2021 as compared to 2019 [3]. According to UNICEF, 67 million children missed out entirely or partially on routine immunization between 2019 and 2021 [4]. Zero-dose children (unvaccinated) increased from 13 million in 2019 to 18 million in 2021 and the partially vaccinated children increased from 6 million in 2019 to 25 million in 2021 [4]. Due to sharp decline in vaccinated children, one in five children worldwide were not fully protected against vaccine-preventable diseases (VPDs). This can lead to secondary outbreaks of VPDs and higher childhood morbidity and mortality. The number of measles cases doubled in 2022 compared with the previous year [5].

Prior to COVID-19 pandemic, inequity in routine immunization has been reported, particularly in low- and middle-income countries (LMICs), with children in low socioeconomic strata and remote rural areas less likely to be fully vaccinated due to inadequate health infrastructure and poor supply chain [6]. Although immunization coverage in India increased from 62% (NFHS 2015-16) to 76.4% (NFHS 2019-21) in children of age 12–23 months by special immunization drives like Mission Indradhanush, there is evidence of existing inequalities in routine immunization coverage in India prior to COVID-19 pandemic [7–10]. Several barriers during the pandemic including parental and health care worker’s concerns regarding exposure to COVID infection, transport restrictions due to lockdown, economic hardships, reallocation of resources, disruptions in supply chains acted as an add-on to the existing inequities in routine immunization [1, 11]. Studies from India have reported major decline in vaccination coverage during the pandemic period [12, 13]. However, no study has been conducted in the post-COVID period to assess the extent of recovery from the impact of the pandemic. We conducted this study to determine the impact of COVID-19 pandemic on routine immunization and assess the immunization status of children in the post-COVID era.

## Materials and methods

We conducted this cross-sectional survey in a tertiary care hospital of Delhi from February 2023 to May 2023 after approval from the institutional ethical committee (IECHR-2023-58-1-R1). According to Kumar et al*.*, 17.8% children admitted in hospital were completely immunized [14]. Sample size was calculated for a power of 80%, and alpha error of 0.05 was 225. Written informed consent were obtained from the parents. Parents of 225 consecutive children 1–6 years in the pediatric ward were interviewed by semi-structured open-ended questionnaire at the time discharge from hospital. Demographic and socioeconomic data were recorded when the children were well enough to be discharged. Children who had received BCG, three doses each of oral polio vaccine (OPV)/pentavalent vaccine/rotavirus vaccine and one dose of measles vaccine within first year of life were classified as completely immunized(14). Those who had missed any dose of a mentioned vaccines were labeled as partially immunized, and those who had not received any vaccine within first year of life were classified as non-immunized(14). Immunization status confirmed with immunization card or hospital prescriptions. Reasons for partial and non-immunization were recorded. If there was delay in routine immunization due to COVID-19 pandemic, reasons were recorded for the same.

Statistical analysis was done using SPSS v29.0 (IBM, SPSS statistics for windows, Armonk, NY: IBM Corp, USA). The *p* value of < 0.05 was considered as significant. Clinicodemographic profile and reasons for delay were expressed as percentages. Number of children with complete, partial and no immunization were expressed as percentage. Association between immunization status and sociodemographic profile was determined using chi-square and logistic regression analysis.

## Results

We enrolled 225 children, 133 (59.1%) were male and 92 (40.9%) females. Age distribution was 92 (40.8%; 95% CI 34–47%) in 12–24 months, 43 (19.1%; 95% CI 14–24%) in 25–36 months and 90 (40%; 95% CI 34–46%) were in 37–72 months age category. Majority of cases i.e., 146 (64.9%; 95% CI 59–71%) resided in Delhi and 79 (35.1%; 95% CI 29–41%) were from neighboring states. Of the 225 children, 162 (72%; 95% CI 66–78%) were completely immunized, 55 (24.4%; 95% CI 19–30%) were partially immunized and 8 (3.6%; 95% CI 1–6%) were unimmunized. For the purpose of analysis, partially and unimmunized cases were grouped together, and characteristics of completely immunized were compared to the combined group of partially and unimmunized cases.

**Fig. 1:**
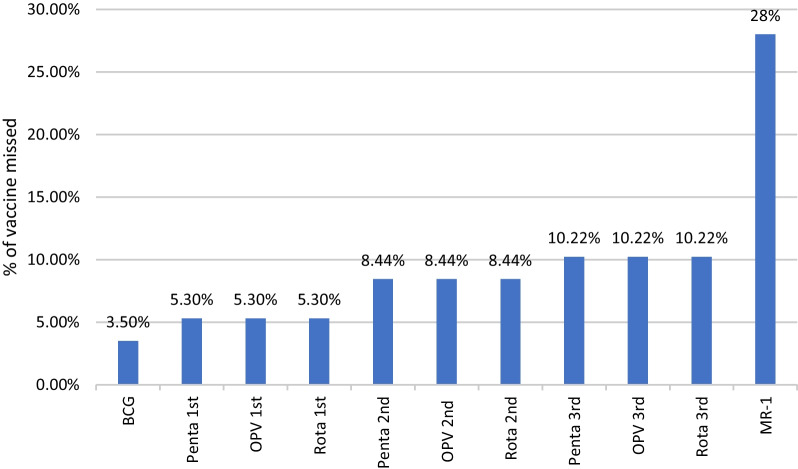
Percentage of vaccine missed by partial/non-immunized children

Table 1 shows the comparison of completely immunized and partial/unimmunized groups in relation to the demographic profiles. Children who had hospital deliveries, birth order ≤ 2 and completely immunized siblings were more likely to be completely immunized (*p* < 0.05). Parents who had better education level were found to have better immunization status of their children (*p* < 0.001). However, sex, address, birth weight, socioeconomic status and distance of vaccination center were not significantly associated with complete immunization (> 0.05). As shown in Fig. 1, 1st dose of measles vaccine and 3rd doses of pentavalent vaccine (DPT + Hib + Hep B), OPV and rotavirus vaccine were most commonly missed vaccine among the partially/non-immunized children.

**Table 1:** Effect of demographic profile on immunization status of children

Sociodemographic factor	Completely immunized (*n* = 162) Number (%; 95% CI)	Partially immunized/unimmunized (*n* = 63) Number (%; 95% CI)	Reference independent variable: AOR^a^ (95% CI)^b^	*P* value
Sex			Male: 0.89 (0.50–1.61)	0.708
Male (*n* = 133)	97 (72.9; 65–81)	36 (27.1; 19–35)		
Female (*n* = 92)	65 (72.0; 61–80)	28 (29.3; 20–39)		
Address			Delhi: 0.69 (0.38–1.26)	0.227
Delhi (*n* = 146)	109 (74.7; 68–82)	37 (25.3; 18–32)		
Outside (*n* = 79)	53 (67.1; 56–78)	26 (32.9; 22–44)		
Place of delivery			Hospital delivery: 0.31 (0.16–0.60)	< 0.001
Hospital (*n* = 175)	136 (77.7; 71–84)	39 (22.3; 16–29)		
Home (*n* = 50)	26 (52.0; 38–66)	24 (48.0; 34–62)		
Birth order			Birth order ≤ 2: 0.46 (0.25–0.84)	0.011
≤ 2 (*n* = 147)	114 (77.6; 71–84)	33 (22.4; 16–29)		
> 2 (*n* = 78)	48 (61.5; 50–73)	30 (38.5; 27–50)		
Birth weight			Birth weight ≤ 2.5 kg: 0.87 (0.48–1.56)	0.64
≤ 2.5 kg (*n* = 127)	93 (73.2; 65–81)	34 (26.8; 19–35)		
> 2.5 kg (*n* = 198)	69 (70.4; 61–80)	69 (29.6; 20–39)		
Immunization status of siblings			Completely immunized sibling: 0.04 (0.01–0.12)	< 0.01
Complete (*n* = 145)	118 (81.4; 75–88)	27 (18.6; 12–25)		
Partial/unimmunized (*n* = 33)	5 (15.2; 2–28)	28 (84.6; 72–98)		
Socioeconomic status			Upper/upper middle/lower middle: 1.14 (0.45–2.92)	0.78
Upper/upper middle/lower middle (*n* = 23)	16 (69.4; 49–90)	7 (30.4; 10–51)		
Upper lower/lower (*n* = 202)	146 (72.3; 66–79)	56 (27.7; 21–34)		
Education of father			Illiterate: 3.30 (1.16–9.40) Primary: 2.60 (0.79–8.57) Middle/high/inter mediate: 1.05 (0.39–2.87) Graduation/professional: 0.58 (0.23–1.50)	0.005
Illiterate (*n* = 67)	36 (53.7; 41–66)	31 (46.3; 34–59)		
Primary (*n* = 28)	19 (67.9; 49–86)	9 (32.1; 14–51)		
Middle/high/Intermediate (*n* = 105)	85 (81.0; 73–89)	20 (19.0; 11–27)		
Graduation/professional (*n* = 25)	22 (88.0; 74–100)	3 (12.0; 2–26)		
Education of mother			Illiterate: 6.31 (1.72–23.13) Primary: 3.47 (0.82–14.71) Middle/high/intermediate: 1.73 (0.47–6.34)	< 0.01
Graduation/professsional: 0.32 (0.09–1.10)				
Illiterate (*n* = 52)	29 (55.8; 42–70)	23 (44.2; 30–58)		
Primary (*n* = 26)	16 (61.5; 41–82)	10 (38.5; 18–59)		
Middle/high/Intermediate (*n* = 116)	91 (79.8; 72–87)	23 (20.2; 13–28)		
Graduation/professional (*n* = 31)	25 (80.0; 66–95)	6 (19.4; 5–34)		

^a^AOR-Adjusted odds ratio

^b^Partially/unimmunized cases is the dependent variable

Reasons for non-immunization and partial immunizations are shown in Table 2. Lack of knowledge and awareness (*n* = 36, 57.1%; 95% CI 45–70%), presence of illness in child (*n* = 21, 33.3%; 95% CI 21–45%) were the common reasons for partial and non-immunization. Lack of knowledge and awareness (50.9%; 95% CI 37–65%) was the most common reason for partial immunization. Other reasons were: busy schedule of parents (*n* = 5), went to village/hometown (*n* = 5), due to religious belief (*n* = 4), disharmony in parents/family (*n* = 1), fear of side effect/reaction/ pain (*n* = 1) and due to shifting of home (*n* = 1). Lack of knowledge and awareness was more common in parents with lesser level of education and in those who resided outside Delhi (*p* < 0.001). However, no association was found between lack of knowledge/ awareness and socio-economic status of the family (*p* > 0.05).

**Table 2:** Reasons for partial and non-immunization

Reason	Partially immunized No. of cases (*n* = 55) (%;95% CI)	Unimmunized No. of cases (*n* = 8) (%; 95% CI)	Total (*n* = 63) (%; 95% CI)
Lack of knowledge and awareness	28 (50.9%; 37–65)	8 (100%)	36 (57.1%; 45–70)
Due to illness	21 (38.2%; 25–51)	0	21 (33.3%; 21–45)
Others	12 (21.8%; 11–33)	5 (62.5%; 19–100)	17 (26.9%; 16–38)
Delay due to covid	11 (20%; 9–31)	0	11 (17.4%; 8–27)

**Fig. 2:**
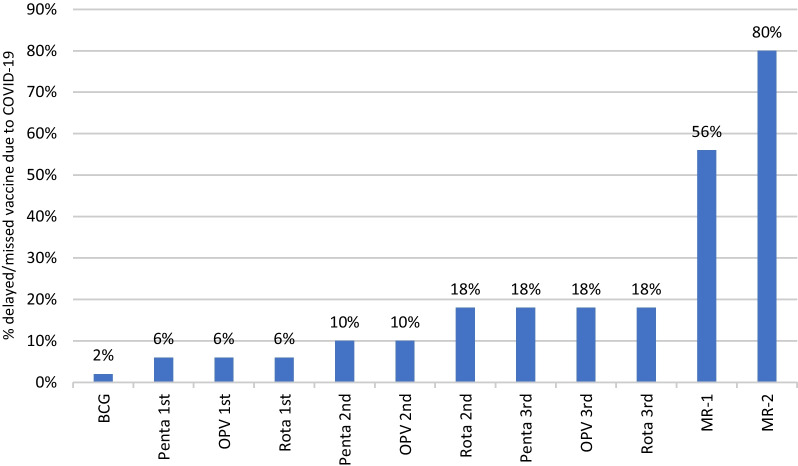
Delayed/missed vaccine due to COVID-19

Delay in immunization due to COVID-19 was found in 50 cases (22.2%; 95% CI 17–28%). Out of these 39 (17.3%; 95% CI 12–22%) had missed their scheduled routine immunization but received vaccination in catch-up visits and were completely immunized at the time of interview. Rest of the 11 cases remained partially immunized. No case remained completely unimmunized due to COVID-19 pandemic. Table 3 shows the reasons for delay in covid-19 pandemic. Restrictions of movement (64%; 95% CI 50–78%), fear of being exposed to COVID-19 (52%; 95% CI 38–66%) were the most common reasons for delay in immunization during COVID-19 pandemic. Others reasons for delay in covid were: non-availability of vaccine in center (*n* = 7), went to village/hometown due to covid pandemic (*n* = 5), nearby vaccination center was closed (*n* = 3), health workers not coming for vaccination (*n* = 2) and someone from the family got ill/died during covid (*n* = 1). As shown in Fig. 1, measles vaccine (MR-1 and MR-2) and 3rd doses of pentavalent vaccine, OPV and rotavirus vaccine were the most commonly missed/delayed vaccine due to COVID-19 pandemic (Fig. 2).

**Table 3:** Reasons for delay in immunization due to COVID-19 (*n* = 50)

Reason	No. of cases (%; 95% CI)
Restrictions on movement	32 (64%; 50–78)
Fear of being exposed to covid-19	26 (52%; 38–66)
Reluctance to leave home	20 (40%; 26–54)
Transport interruptions	16 (32%; 19–45)
Economic hardships	10 (20%; 9–31)
Others	18 (36%; 22–50)

## Discussion

The present study assessed the impact of COVID-19 pandemic on the routine immunization. Our results suggest that COVID-19 pandemic led to delayed vaccinations and disruptions in routine immunization. In our study, 22.2% reported delay in routine immunization due to the pandemic. Our results are consistent with the previous reports. Alsuhaibani et al. reported delay in routine immunization in 24% of cases [15]. A recent study from Rajasthan also reported 31% COVID-19 related disruptions in immunization [16]. Immunization began to decline in March–April 2020 when lockdown was implemented in India. A study from India by Shet et al. reported a decline of 83.1% in vaccination in April–June 2020 and a decline of 32.6% in September 2020 [13]. Although Government of India continued all health services including immunization services during the lockdown but utilization of immunization services substantially declined. Shet et al. reported 33.4% complete or partial suspension of immunization services at various centers in India [13]. Barriers from supply chain and barriers from the caregiver’s side were responsible for decreased utilization of health care services [11]. Consistent with previous research reports, our study showed that fear of contracting COVID-19 was the most common reason among parents [15, 17, 18]. The other reasons included non-availability of vaccines and closed dispensaries/clinics. During the pandemic, many hospitals were providing COVID-19 related services exclusively. During the pandemic, parents preferred home vaccination or facilities dedicated exclusively for immunization services. *Anganwadi* centers (community level mother–child nutrition and welfare centers) which are alternative vaccination facilities were also closed [19]. In our study, few caregivers reported health workers were not coming for vaccination visits as the reason for delay. Health care workers faced challenges in delivering services due to fear of infection, stress, inadequate protective equipment [11]. We found that third dose of pentavalent (DPT3 + Hep B + Hib), OPV and first dose of measles were the most common among missed vaccinations. This finding is consistent with previous studies [20, 21]. In our study, vaccination administration around birth (BCG, hepatitis B) was less affected when compared to vaccination in later stage of life. A likely explanation for this finding is that these children were in health facilities and within the reach of routine health services and the subsequent visits were more affected [22]. Similar trends where administration of BCG and Hepatitis B declined less than later vaccines [12]. On the contrary, Pakistan’s Sindh province reported BCG vaccination as more disrupted than follow-up vaccines due to enrollment services getting more affected [11].

Prevalence of completely immunized children in our study was 72%, and that of partially immunized and non-immunized children were 24.4% and 3.6%, respectively. Studies from pre-COVID era has reported even lower prevalence of fully immunized children. Kumar et al. reported prevalence of fully immunized children as 17.85% [14]. Vohra et al. reported that 56.4% children aged 12–23 months were fully immunized [23]. According to National Family Health Survey (NFHS) 2015–16, 62% children aged 12–23 months were fully vaccinated, whereas NFHS 2019–21 reported 76.4% children as fully vaccinated [7, 8]. These findings show that India has been successful in increasing the immunization coverage with the help of special immunization campaigns such as Mission Indradhanush and supplementary immunization activities such as measles-rubella campaign. Most of the children who had missed vaccination due to COVID-19 pandemic, received their missed vaccination in subsequent catch-up visits.

In the pre-COVID era, the pre-existing inequalities and socio-demographic factors such as parental education, socio-economic class, birth order, religion, family structure have been the challenging factors in achieving the goal of universal immunization. We found that lack of knowledge, child’s illness, busy parents, fear of side effects, religious beliefs were the common reasons for partial and non-immunization. Similarly, Kumar et al. and Vohra et al. documented lack of knowledge, fear of side effects, child’s illness and busy parents as the most common reasons for partial or non-immunization in the pre-COVID era [14, 23]. In the present study, higher parental education, lower birth order and institutional deliveries were found to be associated better immunization status of children. Similar findings have been documented in the previous studies conducted before COVID-19 pandemic [10, 14, 23]. These preexisting challenges were compounded by the disruptions during the COVID-19 pandemic.

The sample size calculation based on 17.8% prevalence of admitted children who are completely Immunized being based on the hospital data does not reflect the true picture and is not representative of the community, this being limitation of the study. This affects generalizability of the study for community. It is noteworthy that a major proportion of the delay/non-immunization was attributed to lack of awareness only a a small chunk was contributed to reverse migration to native villages and non-accessibility of the services in their native places.

## Conclusion

We conclude from our study that although disruptions in COVID-19 pandemic resulted in delay or missed vaccination during the lockdown but to some extent, immunization coverage recovered with catch-up campaigns. Further, immunization coverage more largely depends on the people’s behavior, lack of awareness and socio-demographic factors and not covid pandemic as such. In the future with possibility of similar pandemics, disruptions can be avoided with better resource management and immunization strategies. Recovery in immunization coverage can be achieved with more supplementary immunization activities and awareness campaigns.

## Data Availability

Data and material will be available with Dr Anju Aggarwal.
